# Oral Health as a Risk Factor for Alzheimer Disease

**DOI:** 10.14283/jpad.2023.82

**Published:** 2023-06-28

**Authors:** S. M. Pruntel, B. C. van Munster, J. J. de Vries, A. Vissink, Anita Visser

**Affiliations:** 1 Department of Oral and Maxillofacial Surgery, University Medical Center Groningen and University of Groningen, Groningen, The Netherlands; 2 Department of Internal Medicine, University Medical Center Groningen and University of Groningen, Groningen, The Netherlands; 3 Department of Neurology, University Medical Center Groningen and University of Groningen, Groningen, The Netherlands; 4 Department of Gerodontology, Center for Dentistry and Oral Hygiene, University Medical Center Groningen and University of Groningen, Antonius Deusinglaan 1, Groningen, Groningen, 9713 AV The Netherlands; 5 Department of Gerodontology, College of Dental Sciences, Radboud University Nijmegen Medical Center, Nijmegen, The Netherlands

**Keywords:** Alzheimer’s disease, oral health, cognitive decline, periodontitis

## Abstract

In patients with Alzheimer’s disease pathophysiological changes of the brain that initiate the onset of Alzheimer’s disease include accumulation of amyloid-β plaques and phosphorylation of tau-tangles. A rather recently considered risk factor for the onset of Alzheimer’s disease is poor oral health. The aim of this systematic review of the literature was to assess the potential association(s) of oral health as a risk factor for the onset of Alzheimer’s disease. After a systematic search of Pubmed, Embase and Web of Science. A total of 1962 studies were assessed, of which 17 studies demonstrated possible associations between oral health diseases and Alzheimer’s disease. 4 theories could be distinguished that describe the possible links between oral health and the development or onset of Alzheimer’s disease; 1) role of pathogens, 2) role of inflammatory mediators, 3) role of APOE alleles and 4) role of Aβ peptide. The main common denominator of all the theories is the neuroinflammation due to poor oral health. Yet, there is insufficient evidence to prove a link due to the diversity of the designs used and the quality of the study design of the included studies. Therefore, further research is needed to find causal links between oral health and neuroinflammation that possibly can lead to the onset of Alzheimer’s disease with the future intention to prevent cognitive decline by better dental care.

## Introduction

**A**lzheimer’s disease (AD) is a neurodegenerative disease and the most common cause of dementia. AD accounts for 50–75% of all causes of dementia (1–4). Dementia is the fastest-growing cause of death in the Netherlands (5) and considered as the disease with the most negative impact on quality of life of patients and their caregivers and high costs. Worldwide the total costs for dementia care are around 10 billion euros annually (5). Multiple clinical manifestations can be seen in patients with AD including severe memory loss, problems with spatial recognition, and impaired reasoning or judgment (6’^8^). The course of AD is slow and irreversible, and finally leads to death (9, 10). In literature a diversity of physiological changes in the brain has been described and several risk factors for the onset of AD have been discovered, however the cause of AD and the precise pathway to it’s onset are still not fully known (5, 11).

Pathophysiological changes of the brain of AD patients include accumulation of amyloid-β plaques (Aβ) and phosphorylation of tau-tangles (12’^14^). These changes are considered to be the most important pathological causes for AD as they show a specific spatial and temporal dispersion resulting in degeneration of the affected cerebral cortices (13, 15). Other pathological processes, such as cerebrovascular abnormalities, inflammation and oxidative stress, have also been shown as contributors to AD pathogenesis in imaging and obduction studies (16). It is believed that the buildup and deposition of Aβ in the brain occurs early in the course of the disease and sets off subsequent processes such as tau phosphorylation, inflammation, and oxidative stress that all result in neurodegeneration (17). Several risk factors for AD are known such as an unhealthy life style, malnutrition, brain injury, low education attainment, depression, social isolation, cognitive inactivity and air pollution (18–21).

A rather recently considered risk factor for the onset of AD is poor oral health (22–24). It has been reported that poor oral health is often seen in patients with AD (23, 24). This phenomenon might be the result of cognitive decline which can affect oral hygiene routines and dental visits (25, 26), but it has also been suggested that oral health illness, in particular periodontitis, is a risk factor for a exacerbation of the neuroinflammation that, e.g., could result in a chronically elevated proinflammatory status. This status could lead to neurodegeneration through various pathways (27–29). It already has been shown that a systemic effect of oral illness on general health may be caused by the invasion of oral pathogens into the airway (e.g., due to dysphagia) and or the bloodstream (e.g., via the periodontal tissues) (30). Given the impact of AD on patients, their caretakers and society, it is important to prevent the onset of AD where ever possible. Oral health in particular periodontal disease is preventable and treatable. Therefore, the aim of this systematic review of the literature was to assess the potential association(s) of oral health as a risk factor for the onset of AD.

## Methods

This systematic review is reported according to the PRISMA statement to ensure quality and completeness. This study’s protocol was registered in PROSPERO prior to the systematic literature search (registration number CRD42023402291) (31).

### Search strategy

Pubmed, Embase and Web of Science databases were searched. Search terms were: Alzheimer’s disease, periodontal disease, caries, dental infections, mobile teeth, loss of teeth, periodontitis, gingivitis, dental health, oral health, hyposalivation, xerostomia, oral pain, broken teeth, dry mouth, and chewing problems. For the complete search strategy see Table 1. The literature search was searched on 01-01-2023. The sensitive search strategy was developed together with an information specialist from the university library (Karin Sijtsma) and adapted appropriately to each database.

**Table 1:** Search terms in Pubmed, Embase and Web of Science

**Pubmed:**	**Embase:**	**Web of Science:**
(«Alzheimer Disease»[Mesh] OR Alzheimer*[tiab]) AND («Periodontal Diseases»[Mesh] OR «Tooth Diseases»[Mesh] OR «Oral Health»[Mesh] OR «Xerostomia»[Mesh] OR «Halitosis»[Mesh] OR «Dental Care»[Mesh] OR periodon*[tiab] OR caries*[tiab] OR dental*[tiab] OR dentist*[tiab] OR teeth*[tiab] OR tooth*[tiab] OR oral health*[tiab] OR gingiv*[tiab] OR hyposalivation*[tiab]	(‘Alzheimer disease’/exp OR (Alzheimer*):ab,ti,kw) AND (‘tooth disease’/exp OR ‘halitosis’/exp OR ‘xerostomia’/exp OR’ dental procedure’/exp OR ‘mouth hygiene’/exp OR (periodon* OR caries* OR dental* OR dentist* OR teeth* OR tooth* OR ‘oral health*’ OR gingiv* OR hyposalivation* OR xerostomia* OR ‘chewing problems*’ OR masticat* OR halitosis*):ab,ti,kw) NOT	TS=(Alzheimer*) AND TS=(periodon* OR caries* OR dental* OR dentist* OR teeth* OR tooth* OR “oral health*” OR gingiv* OR hyposalivation* OR xerostomia* OR “chewing problems*” OR masticat* OR halitosis*) NOT TI=(rat OR rats OR animal OR mouse OR mice)
OR xerostomia*[tiab] OR chewing problems*[tiab] OR masticat*[tiab] OR halitosis*[tiab]) NOT	((‘animal’/exp OR ‘human’/exp) OR (rat OR rats OR animal OR mouse OR mice):ti)	
((«Animals»[Mesh] NOT «Humans»[Mesh]) OR rat[ti] OR rats[ti] OR animal[ti] OR mouse[ti] OR mice[ti])		
742 articles, 1 January 2023	55 articles, 1 January 2023	1165 articles, 1 January 2023

### Study selection

Studies were included when they reported on the potential association of oral health as a risk factor for the onset of AD. Excluded were articles reporting on animals, cellular level, case reports, review articles, letters to the editors and trial designs. There were no exclusions based on study design or language.

First, duplicates found in Pubmed, Embase and Web of Science were removed. Titles and abstracts were screened for selection by two observers independently (SP and AnV). Articles with a title that had insufficient information and/or a lacking abstract were screened by full text assessment. If an abstract provided insufficient information or disagreement existed between observers, the full text was also checked. If no full text was available or if parts of the study were unclear, the authors of the study were contacted for additional information. The reference sections of all included and review articles in the full text phase were scrutinized for additional articles. Librarians in the university were able to provide translations when needed.

### Quality assessment

The quality of the studies was analyzed by SP and AnV based on the Methodological Index for Non-Randomized Studies (MINORS) (32). 32 contains 12 methodological points, the first eight apply to both non-comparative and comparative studies, while the remaining four relate only to studies with two or more groups. The items were scored 0 if not reported; 1 when reported but inadequate; and 2 when reported and adequate. The ideal score is 16 for non-comparative studies and 24 for comparative studies. MINORS is a valid instrument designed to assess the methodological quality of non-randomized studies, whether comparative or non-comparative.

## Results

### Search result

**Figure 1:**
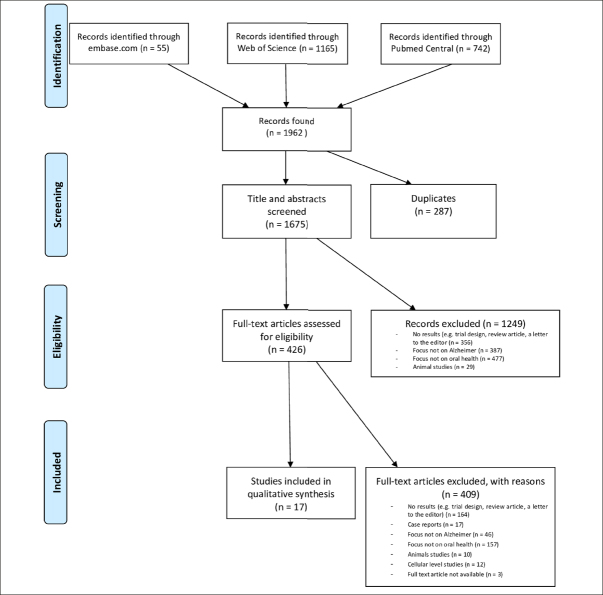
Flow diagram of the study process and article selection

The database search resulted in 1962 hits. After removing duplicates, 1675 unique papers remained (shown in Fig. 1). After title and abstract screening 426 articles remained of which 409 were excluded after full text reading. Hence 17 studies could be included for quality / risk of bias assessment and data extraction.

### Study characteristics

All included papers could be characterized as prospective and or cross sectional studies describing different aspects of oral health as potential factors for the development of AD (table 2). As shown in table 2, there was considerable clinical heterogeneity in sample size, age and design. The number of included patients in the studies varied from 14 to 309 with a total of 1807 patients. Reported age differed between the studies, with a mean age of 72 years (range 40–104). The mean age of the AD groups were 74,5 and control groups were 70,4. In one study, age was not described (33). The mean follow-up was 6.8 years (range: 0.5–10). The included studies could be categorized according to 1) role of pathogens, 2) role of inflammatory mediators, 3) role of APOE alleles and 4) role of Aβ peptide (Table 2). According to MINORS, the quality of most included studies was reasonable to good (Table 3).

**Table 2:** Characteristics of the included studies

**Study**	**Study design**	**Aim**	**Population**	**Gender, M (%)**	**Age, mean (SD)-yrs**	**Outcomes**	**MINORS score**	**Follow-up (years)**
**Role of oral pathogens**								
Panzarella et al. 2020(39)	Cross-sectional	To evaluate the oral health status and its relationship with cognitive impairment of participants, enrolled in the Zabut Aging Project, a community-based cohort study performed in rural community in Sicily, Italy.	60 (20 AD, 20 MCI and 20 controls)	AD 12 (60)	AD 83.5 (7.7)	The decayed, missing, and filled teeth (DMFT) total score of AD subjects was significantly higher than for a MCI and controls. Furthermore, the «M» component of DMFT (i.e., the number of missing teeth) was significantly higher in AD than in a MCI and controls. A Poisson regression model revealed that age, male gender, and AD were positively correlated with DMFT. Concerning oral microbial load, the presence of Fusobacterium nucleatum was significantly higher in AD than in controls, and a higher load of Treponema denticola was found in aMCI than with AD. OHR-QoL scores did not differ among the groups.	20	10
Leblhuber et al. 2019(34)	Cross-sectional	To find a link exist between oral pathogens and Alzheimer’s disease.	20 AD	11 (55)	78.1(2.2)	The presence of Porphyromonas gingivalis was found to be associated with lower mini mental state examination scores and with a tendency to lower scores in the clock drawing test. Furthermore, association between lower serum concentrations of the immune biomarker neopterin and the presence of Treponema denticola as well as of kynurenine were found in Alzheimer patients positive vs. negative for Tannerella forsytia. Six of the patients positive for periodontal pathogens were ApoE4 (apolipoprotein E4) allele carriers, two of them homozygous.	5	NA
Stein et al. 2012(41)	Prospective	To examine serum antibody levels to bacteria of periodontal disease in participants who eventually converted to AD compared with the antibody levels in control subjects.	158 (35 AD, 46 MCI and 77 controls)	9 (26%)	74.1 (7.5)	Antibody levels to F nucleatum and P. intermedia were significantly increased (a<0.05) serum draw in the patients with AD compared with controls. These results remained significant when controlling for baseline age, Mini-Mental State Examination score, and apolipoprotein epsilon 4 status.	20	11,7
Riviere et al. 2002(33)	Prospective	The purpose of this investigation was to use molecular and immunological techniques to determine whether oral Treponema infected the human brain.	34 (16 AD and 18 controls)	NA	NA	CR detected Treponema in 14/16 Alzheimer’s disease (AD) and 4/18 non-AD donors, and AD specimens had more Treponema species than controls. PCR also detected Treponema in trigeminal ganglia from three AD and two control donors. Cortex from 15/16 AD subjects and 6/18 controls contained Treponema pectinovorum and /or Treponema socranskii species-specific antigens. T. pectinovorum and /or T. socranskii antigens were also found in trigeminal ganglia and pons from four embalmed cadavers, and 2/4 cadavers also had Treponema in the hippocampus.	14	NA
Wu et al. 2021(38)	Prospective	To evaluate sequence to determine the relative abundance and diversity of bacterial taxa in the dental plaque of elderly patients with AD and controls.	35 (17 AD and 18 controls)	6(35)	77.9 (10.5)	Significantly increased numbers of Lactobacillales, Streptococcaceae, and Firmicutes/ Bacteroidetes and a significantly decreased number of Fusobacterium were observed in patients with AD.	16	NA
Holmer et al. 2021(40)	Cross-sectional	To compare the subgingival microbiota of people with AD, MCI, subjective cognitive decline (SCD) and cognitively healthy individuals.	132 (46 AD, 40 MCI, 46 subjective cognitive decline (SCD), and 63 controls)	23 (50)	71 (11)	Two Operational Taxonomic Units were particularly abundant in AD compared to controls: Slackia exigua, which was also associated with deep periodontal pockets, and a Lachnospiraceae [G-7] bacterium.	18	NA
Laugisch et al. 2018(37)	Cross-sectional	Verification of the presence of periodontal pathogens and the intrathecal generation of pathogen-specific antibodies in 20 patients with AD and 20 with other forms of dementia (DEM-noAD).	40 (20 AD and 20 other forms of dementia(DEM-noAD))	9 (45)	58.3 (5.3)	In line with diagnoses, CSF-levels of A1-42 were significantly lower in AD than DEM-noAD patients. Periodontal destruction and inflammation were omnipresent with no difference between groups. P. gingivalis, T. forsythia, and Treponema species were detected in more than 50% of subgingival biofilm samples, but neither in serum nor in the CSF. Elevated levels of anti-pathogen antibodies in CSF of 16 patients (7 AD; 9 DEM-noAD) compared to serum highlight a possibility of the intrathecal immune response to pathogens. There was no significant difference in antibodies levels against selected bacteria in CSF and serum between groups. Multivariate regression analysis and general linear models revealed an association of the T-tau level in AD group with both serum levels of anti-P. gingivalis antibodies and MCP-1/CCL-2.	18	NA
Noble et al. 2014(35)	Prospective	To study serum IgG to periodontal microbiota as possible predictors of incident AD.	219 (110 AD and 109 controls)	35 (31.8)	78.9 (7.2)	In a model adjusting for age, sex, education, diabetes mellitus, hypertension, smoking, prior history of stroke, and apolipoprotein E genotype, high anti-A.- naeslundii titer (0.640 ng/ml, present in 10% of subjects) was associated with increased risk of AD (HR52.0, 95%CI: 1.1–3.8). This association was stronger after adjusting for other significant titers (HR53.1, 95%CI: 1.5–6.4). In this model, high anti-E. nodatum IgG (.1755 ng/ ml; 19% of subjects) was associated with lower risk of AD (HR50.5, 95%CI: 0.2–0.9).	22	5
**Role of inflammatory mediators**								
Cestari et al. 2016(49)	Prospective	To investigate the prevalence of oral infections and serum levels of IL-6 and TNF-a in patients with AD, MCI, and non-demented elderly.	65 (25 AD, 19 MCI and 21 controls)	10 (40%)	63–92 years	Patients with AD had high serum IL-6 levels, and patients with periodontitis had high serum TNF-a levels. There was an association between IL-6 and TNF-a in patients with AD/MCI and periodontitis.	16	NA
Farhad et al. 2014(46)	Prospective	To evaluate the effect of chronic periodontitis on serum levels of tumor necrosis factor-α in Alzheimer disease.	80 AD (40 chronic periodontitis and 40 controls)	NA	40 and 70 years	It showed that the mean of tumor necrosis factor-α in patients with Alzheimer and periodontitis was approximately three folds higher than the patients only with Alzheimer, and this difference was statistically significant.	20	NA
Ide et al. 2016(42)	Prospective	To determine if periodontitis in Alzheimer’s disease is associated with both increased dementia severity and cognitive decline, and an increased systemic pro inflammatory state.	59 (22 AD with periodontitis and 37 AD without periodontitis)	13 (59%)	74.9 (2.0)	The presence of periodontitis at baseline was not related to baseline cognitive state but was associated with a six fold increase in the rate of cognitive decline as assessed by the ADAS-cog over a six month follow up period. Periodontitis at baseline was associated with a relative increase in the pro-inflammatory state over the six month follow up period.	20	0,5
Gil-Montoya et al. 2020(48)	Prospective	To examine the impact of inflammation on the relationship between periodontitis and cognitive impairment.	309 (156 AD, 22 MCI and 131 controls)	59 (33)	77.1 (7.8)	At the time of sampling, 11 of the 29 inflammatory biomarkers were associated with cognitive impairment in patients with more severe periodontitis. However, the inflammatory response to severe periodontitis was more reduced (lower biomarker concentrations) in cases (with cognitive impairment or dementia) than in (cognitively healthy) controls, an unexpected finding.	18	NA
Kamer et al. 2009(47)	Prospective	To hypothesize that AD patients would have both an elevated cytokine expression and positive antibodies to P. gingivalis, T. forsythia and A. actinomycetem-comitans, and that these measures would contribute to clinical separation of AD from cognitively normal subjects.	34 (18 AD and 16 controls)	4 (22)	40-80+ years	Plasma TNF-a and antibodies against periodontal bacteria werl?e elevated in AD patients compared with controls and independently associated with AD. The number(47) of positive IgG to periodontal bacteria incremented the TNF-a classification of clinical AD and controls.	18	NA
**Role of APOE-4 allels**								
Popovac et al. 2020(52)	Prospective	To examine the impact of dental status and different APOE gene variants on AD occurrence. Secondly, socio demo graphic variables were investigated as factors potentially associated with AD.	179 (116 AD and 27 controls)	39 (33.6)	65–85+ years	Number of total FTU and presence of APOE4 allele remained significant as independent risk factors for AD even when adjusted for age, sex, and level of education.	16	NA
Popovac et al. 2016(53)	Cross-sectional	To determine the frequency of APOE alleles and their association with the dental status in elderly demented patients.	67 AD	19 (28.4)	65 or older	DNA was isolated from buccal swabs and genotyping was done by PCR-RFLP. The majority of participants had E3/E4 genotype (55.2%) and these heterozygotes were significantly more frequent than any other genotype (p<0.001).	8	NA
**Role of Amyloid B peptide**								
Kubota et al. 2014(54)	Prospective	To analyse the expression levels of ABP, IL-lb, and C1QA and determined the localisation of ABP in gingival tissues.	28 (14 periodontitis patients and 14 controls)	7 (50%)	58.0 (16.0)	ABP, IL-lb, and C1QA mRNA levels were significantly up regulated in periodontitis affected gingival tissues. ABP was mainly localized in macrophages in gingival connective tissues underneath the epithelial layers.	15	NA
Gil-Montoya et al. 2017(55)	Prospective	To determine whether periodontitis is related to the amyloid b (Ab) load in blood and the role of any such relationship in the association between ABP and cognitive impairment.	288 (166 cognitive impairment with or without AD and 122 controls)	57 (34.3)	77.3 (7.8)	Higher blood Ab1–42 levels (P = 0.01) and higher Ab42:40 ratio (P = 0.06) were observed in participants with severe attachment loss than in other participants. Periodontitis was a significant interaction variable, given that the association between Ab1–42 and Ab1–40 and cognitive impairment was only observed in patients with severe periodontitis. According to these data, periodontitis may be a modulating variable of the association between ABP and cognitive impairment.	16	NA

**Table 3:** MINORS score (0 if not reported; 1 when reported but inadequate; and 2 when reported and adequate). The ideal score is 16 for non-comparative studies and 24 for comparative studies

	**A clearly stated aim**	**Inclusion of consecutive patients**	**Prospective collection of data**	**Endpoint appropriate to the aim of the study**	**Unbiased assessment of the study endpoint**	**Follow-up period appropriate to the aim** **of the study**	**Loss of follow up less than 5%**	**Prospective calculation of the study size**	**An adequate control group**	**Contemporary group**	**Baseline equivalent of groups**	**Adequate statistical analysis**	**Total**
Panzarella et al. 2020	2	2	0	2	0	2	2	0	2	2	2	2	18/24
Leblhuber et al. 2019	2	2	0	2	0	0	0	0	0	0	0	0	6/16
Cestari et al. 2016	2	2	2	2	0	0	0	0	2	2	2	2	16/24
Farhad et al. 2013	2	2	2	2	0	0	0	0	2	2	2	2	16/24
Cestari et al. 2016	2	2	2	1	0	0	0	0	2	2	2	2	15/24
Stein et al. 2012	2	2	2	2	0	2	2	0	2	2	2	2	20/24
Kamer et al. 2009	2	2	2	2	0	0	2	0	2	2	2	2	18/24
Riviere et al. 2002	2	0	2	2	0	0	0	0	2	2	2	2	14/24
Wu et al. 2021	2	2	2	2	0	0	0	0	2	2	2	2	16/24
Holmer et al. 2021	2	2	0	2	0	0	2	0	2	2	2	2	16/24
Popovac et al. 2021	2	2	2	2	0	0	0	0	2	2	2	2	16/24
Montoya et al. 2019	2	2	2	2	0	0	0	2	2	2	2	2	18/24
Laugischs et al. 2018	2	2	0	2	0	0	0	2	2	2	2	2	16/24
Montoya et al. 2017	2	2	2	2	0	0	0	0	2	2	2	2	16/24
Ide et al. 2017	2	2	2	2	0	2	0	2	2	2	2	2	20/24
Popovac et al. 2016	2	2	0	2	0	0	0	0	0	0	0	0	6/16
Noble et al. 2014	2	2	2	2	0	2	2	2	2	2	2	2	22/24

### The role of oral pathogens

Eight out of the 17 studies described associations between oral pathogens and AD (33–41). Multiple studies showed that oral bacterial species (e.g., Fusobacterium nucleatum and Prevotella intermedia), who are known to be involved in the development of periodontitis, were found in higher percentages in AD patients than in non-AD patients (34, 36, 39, 41, 42). In the study of Stein et al. (41) antibody levels towards the periodontal bacteria P. intermedia and F. nucleatum were significantly increased at baseline in serum in patients with AD when compared to controls. In this study the response to periodontal bacteria in AD patients years before cognitive impairment suggests that the bacterial load of periodontal disease could potentially contribute to the risk of the development of AD (41). The median time from baseline assessment to diagnosis for the AD subjects was 9.6 years (41).

Maurer et al. investigated a possible link between bacterial infestation of the mouth, oral health and AD (43). They discovered that resistant oral bacteria (A. actinomycetemcomitans, P. gingivalis, and F. nucleatum) were present on the surface of the maxillary molars in AD patients. These bacteria formed a complex biofilm. The authors hypothesized that bacterial toxins may cause inflammatory processes that trespasses neighboring parts of the central nervous system that are associated with the onset of AD such as the entorhinal cortex and the hippocampus. This might also explain the loss of smell in AD patients (entorhinal cortex) (43). Laugisch et al. concluded from their studies that periodontal pathogens could enter the brain and stimulate a local immune response, however, their data does not support a specific association of periodontal infection with an onset of AD in an age up to 70-year-old patients and early stages of the disease (37). Similar levels of periodontal infection were found in patients with AD and other forms of dementia (37).

Two post-mortem studies from Riviere et al. and Poole et al. compared the microbiome of the brain tissue of AD patients to that of cognitively normal patients (33, 44). Rivière et al. found in the frontal lobe cortex that treponemes (T. amylovorum, T. denticola, T. maltophilum, T. medium, T. pectinovorum, T. socranskii, T. vincentii) which are associated with periodontitis colonized the brain more frequently in AD patients than in controls (33). Next, Poole et al. found P. gingivalis (pg)- lipopolysaccharides (LPS) in early post-mortem and proposed that pg-LPS have a role in brain inflammation associated with AD (45). In non-AD control tissues, no evidence of pg-LPS was seen (45). This study confirms that in AD patients, LPS from periodontal bacteria can access the brain during life (45).

### Role of inflammatory mediators

Five studies assessed the role of oral inflammatory mediators originating from the mouth in patients with AD(46–49). Pro-inflammatory cytokines (interleukin 1(IL-1), interleukin-6 (IL-6) and tumor necrosis factor-α (TNF-α)) might be a connecting link between poor oral health and AD as these cytokines can penetrate the blood-brain barrier and activate the resident microglial cells (49). Human gingival fibroblasts (HGFs) are the most abundant cells in gingival connective tissues (50). Various HGF responses to periodontal pathogens or inflammatory cytokines contribute to the development of periodontitis. There they can trigger the production of Amyloid Beta Protein (ABP) and Tau phosphorylation, resulting in neuronal damage and cognitive impairment, and thus be involved in AD development (49). IL-6 is an important cytokine involved in the regulation of host response to bacterial infection (50). IL-6 levels in AD patients were substantially higher than in controls, and periodontitis patients had noticeably higher TNF-a levels than AD patients with healthy periodontium (49). In the AD and mild cognitive impairment (MCI) groups, IL-6 and TNF-a were likewise positively associated (49). Thus, patients with AD and poor periodontal health had greater TNF-α levels in their blood compared with healthy controls (49).

Multiple studies found that the levels of TNF-α were significantly increased in AD subjects compared to the controls, while IL-1β and IL-6 levels did not differ between AD subjects and controls (47, 49). These findings suggest that antibodies against periodontal bacteria are linked to AD and may aid in the clinical identification of the disease (47).

The diagnostic signature pointed towards a central role for TNF-α and significant roles for several immune-associated plasma proteins. As antibody response reflects host immune function, we hypothesize that antibody titers to periodontal bacteria may complement plasma TNF-α levels in improving the clinical diagnosis of AD patients and differentiate them from cognitively normal subjects (47, 49).

Ide et al. showed that periodontitis was not associated with baseline cognitive status like onset AD, but periodontitis was linked to a six-fold increase in the rate of cognitive decline throughout a six-month follow-up period (42). A follow up study of Noble et al. studied if IgG to periodontal microbiota as possible predictors of AD (35). They showed that not only IgG levels are associated with risk for developing AD, but also an increase of IgG levels in the time without an obvious cause (35). Both were not found in the control group.

### Role of APOE-4 allels

Bergdahl et al. found that the edentate patients (age mean 72 years) had a higher ApoE4 in the AD patients than dentate AD patients (age mean 54 years) (51). Also the results of the studies of Popovac et al. showed that presence of ApoE4 allele and low number of functional tooth units may independently both raise the risk of AD (52, 53). The few studies that looked at dental and genetic factors as predictors of AD showed the association that having at least one ApoE4 allele and fewer than eight teeth increased the likelihood of mild memory impairment, although only the risk factor ApoE4 allele did significantly increase the risk of AD (51). Fewer teeth can be caused by inflammation of the gums, which can be caused by oral pathogens.

### Role of Amyloid Beta Protein

ABP may play a role in the development of AD (52–54). Patients with severe periodontal disease have higher plasma ABP concentrations and the presence of periodontitis may modulate the observed association between ABP and cognitive impairment (55). Kubota et al demonstrated an association between periodontitis and AD using a combination of microarray and computer-aided data mining analyses (54). qRT-PCR successfully identified differential expression of specific genes related to periodontitis and AD pathogenesis (54). Immunolocalisation of APP in gingival tissue was also investigated suggesting a potential mechanism by which chronic periodontitis may be biologically linked to the clinical onset or progression of AD (54).

## Discussion

This systematic review of the literature aimed to assess the potential association of oral health as a risk factor for the onset of AD. Only a few studies demonstrated possible associations between oral health diseases and AD (34). When summarizing these studies, four theories could be distinguished that describe the possible links between oral health and the development or onset of AD as can be read in the results section. These four theories are discussed below. The main common denominator of all the theories is the ‘inflammatory burden that occurs. The theory of the role of Aβ is the least likely and the theory of oral pathogens is the most likely.

### Role of oral pathogens

Some studies suggest that bacteria and their toxins can enter the brain via the central nervous system, and provoke a chain reaction that might lead to AD (42, 56, 57). Several pathways are optional for this theory. In literature the gut–brain axis is a well-known bidirectional link between the gut microbiome and the brain/ central nervous system as has been shown for e.g. Parkinson disease (PD) (42, 56, 57). For PD accumulating evidence suggests that the onset of non-motor symptoms, such as gastrointestinal manifestations, can precedes the onset of motor symptoms and disease diagnosis, supporting a potential role for the microbiome-gut-brain axis in the underlying pathological mechanisms (58–60). This bidirectional relationship is also thought to exist between the oral microbiota and AD (42, 56, 57). Thus, the dysbiotic signature in AD patients particularly with regard to periodontal bacteria, indicates that the mouth bacterial flora and it’s toxins may affect the central nervous system (61). For AD counts that some oral microorganisms and their toxins have already be found to transfer into the brain via cranial nerves, raising the chance of developing AD (57, 61).

F. nucleatum and T. denticola bacterial load in the mouth was significantly higher in AD than controls (39). F. nucleatum is one of the most common species in the human gingival, and may be isolated both from healthy and compromised periodontal teeth, thus the cause between the bacterial load and AD is not yet clear (39, 62). Which pathogens and associated pathways are involved in the development and progression of AD remains to be elucidated, but understanding the specific mechanisms involved in the interaction between these pathogens and the nervous system is vital for the early intervention in AD.

### Role of inflammatory mediators

Neuroinflammation is an important process in AD neurodegeneration by the vicious circle of inflammation and cell destruction (63). The presence of lipopolysaccharides from T. denticola, T. forsythia and P. gingivalis in the brain, which can be associated with increased expression of major proinflammatory cytokines (IL-1β, IL-6 and TNFα) and decreased expression of antiinflammatory cytokines (IL-10), suggest a role for the interaction of inflammatory markers in the development and progression of AD (63, 64). In addition, these periodontal pathogens appear to modulate the immune response in AD patients. This modulation may be related to an alteration in microglial activity, which is associated with an increased risk of AD (64). However, it appears that the oral pathogens and inflammatory mediators amplify a chain reaction, but there is no clear evidence of an association between the mouth inflammatory mediators and AD. More research is needed to understand the relationship with important inflammatory pathways and to assess the detailed interactions between periodontitis and AD.

### Role of APOE-4 allels

The APOE-4 allels appears to play a critical role in neuroinflammation and may contribute to promoting P. gingivalis colonisation of the brain (44, 65, 66). Poole et al. observed the presence of P. gingivalis DNA in the brain of APOE mice which were infected at the gingival level with this gram-positive pathogen (44). Interestingly, as shown by Singhrao et al, gingival infection with P. gingivalis also resulted in the early appearance of age-related granules in these mice (66). These data, in line with the results of another study by Hafezi-Moghadam et al., suggest that the lack of APOE allel and the increased systemic inflammation observed in periodontitis induce an impairment of the blood-brain barrier (65). A larger population is needed to confirm these findings (65). It is impossible to conclude that the APOE-4 allele plays a pivotal role in this complex interaction of oral inflammation and AD based on the findings of the preceding investigations.

### Role of Amyloid Beta Protein

The passage of ABP from the bloodstream to the brain may explain the previous finding of an association between periodontitis and AD and the present observation that increased plasma ABP concentrations are associated with AD and modulated by periodontal disease (67). Periodontitis has been linked to Aβ accumulation in distant brain areas thought to be sensitive to AD (55). According to Montoya et al., patients with severe periodontal disease and AD had higher plasma ABP concentrations, and the presence of periodontitis may affect the ABP-AD relationship (55). The passage of ABP from the bloodstream to the brain may explain a previous finding of an association between periodontitis and AD (55). The involvement of ABP was validated in a study by Kamer et al., who discovered that cognitively healthy persons with periodontal disease had more Aβ accumulation in their brain than those without poor oral health (47). They found that periodontal disease was linked to amyloid buildup in the brain in areas prone to amyloid accumulation in AD patients (47, 55).

All ABP findings imply that poor oral health may enhance the risk of amyloid accumulation in the brain (47). Plasma ABP levels are higher in individuals who have severe periodontal disease (55). Thus, the presence of periodontitis may modify the association between ABP and cognitive impairment (55). Faulty inflammatory mechanisms may play a role in the onset and progression of age-related chronic diseases such as AD, cardiovascular disease, cancer, diabetes and periodontitis (54, 55). Finally, it remains unclear whether increased concentrations of ABP in the brain are responsible for the neurodegeneration and/or the cause of oral health disease. In the above studies, all patients already had AD (54, 55). Therefore, studies are eagerly awaited in patients who do not yet have AD, but develop it later.

### Strengths and limitations

Most of the included studies met the criteria as described in the MINORS qualification (32). The quality of most studies was reasonable to good. Limitations of this systematic review are that the studies differed in study design (cross-sectional and prospective), study protocol, sample sizes, and whether the oral problem was actually present first before the cognitive problem. Furthermore, the definition for oral health in the various studies hampered comparison (39, 68, 69). A proper definition is eagerly awaited. Finally, not all studies compared AD with healthy controls, the mean age in the AD groups was often much higher than the control groups, and in most studies the control group had fewer individuals.

## Conclusion

From this review it can be concluded that four main theories can be distinguished that might give an explanation for all associations found between AD and oral health. Yet, there is insufficient evidence to prove a link between AD and oral health due to the diversity of study designs of the included studies. Therefore, further research is needed to search for causal links between oral health (in particular periodontitis) and neuroinflammation that can lead to the onset of AD with the future intention to prevent cognitive decline by better dental care.

## Data Availability

All data generated or analyzed during this study are included in this article. Further enquiries can be directed to the corresponding author.

## Notes

*Statement of Ethics:* An ethics statement is not applicable because this study is based exclusively on published literature.
